# Prolonged screen watching behavior is associated with high blood pressure among children and adolescents: a systematic review and dose–response meta-analysis

**DOI:** 10.1186/s41043-023-00437-8

**Published:** 2023-08-31

**Authors:** Mahdieh Abbasalizad Farhangi, Elahe Fathi Azar, Ali Manzouri, Fariborz Rashnoo, Amir Shakarami

**Affiliations:** 1 Tabriz Health Services Management Research Center, Tabriz University of Medical Sciences, Tabriz, Iran; 2 Department of Occupational Therapy, School of Rehabilitation Sciences, University of Social Welfare and Rehabilitation Sciences, Tehran, Iran; 3 Health Policy Research Center, Institute of Health, Shiraz University of Medical Sciences, Shiraz, Iran; 4 Department of General and Minimally Invasive Surgery, Loghman Hakim Hospital, Shahid Beheshti University of Medical Sciences, Tehran, Iran; 5 Department of Cardiovascular Medicine, Lorestan University of Medical Sciences, Khorramabad, Iran

**Keywords:** Screen time, Hypertension, Children, Adolescents, Dose–response

## Abstract

**Background:**

Numerous cardio-metabolic risk factors influence screen-related behaviors in children and adolescents. Numerous studies with inconsistent results revealed a relationship between blood pressure and screen time in the children and adolescents. This systematic review and meta-analysis summarized the data regarding the relationship between screen time and hypertension (HTN) in children and adolescents.

**Methods:**

We examined three electronic databases, including Scopus, PubMed, and Embase to find the recent research on the relationship between screen time and HTN up to 19 July 2022. Twenty papers were included in the final two-class and dose–response meta-analysis. We conducted subgrouping to identify the source of heterogeneity.

**Results:**

The highest category of screen time increased the odds of HTN by 8% [odds ratio (OR): 1.15; 95% confidence interval (CI): 1.08, 1.23; *P* < 0.001; *I*^2^ = 83.20%] and 1.9 mmHg increase in systolic blood pressure [weighted mean difference (WMD): 1.89; 95% CI: 0.18–3.62; *P* = 0.030; *I*^2^ = 83.4]. However, there was no significant difference in diastolic blood pressure. Moreover, screen time in hypertensive children and adolescents was 0.79 h (47.4 min) higher than normotensive subjects (WMD: 0.79; 95% CI: 0.02, 1.56; *P* = 0.046; *I*^2^ = 92.8). A departure from linearity was observed between increased screen time [digital video discs, personal computers, and video games and HTN (*P*_nonlinearity_ = 0.049).

**Conclusion:**

This systematic meta-analysis review is the first to demonstrate a positive correlation between screen time and HTN in children and adolescents.

**Supplementary Information:**

The online version contains supplementary material available at 10.1186/s41043-023-00437-8.

## Introduction

Hypertension (HTN) and high blood pressure have been considered major health problems among children and adolescents in recent decades [1]. Both pre-HTN and HTN are on the rise globally. In this respect, the results of a recent meta-analysis conducted on more than 54,196 participants showed that the pooled prevalence of hypertension was 5.5% (95% CI: 4.2–6.9), while the prevalence of slightly increased blood pressure among children and teenagers aged two to19 was 12.7% (95% CI: 2.1–30.4) [2, 3].

HTN in childhood is associated with early cardiovascular events like congestive heart failure (CHF), left ventricular hypertrophy (LVH), and increased morbidity and mortality in adulthood [4, 5]. Affecting more than 2.5–17.3% of adolescents [6], HTN in childhood and adolescence is described to track into adulthood and is associated with major cardiovascular problems such as heart failure, stroke, and congestive heart disease [1, 7]. In recent years, the increasing prevalence of HTN among children and adolescents in numerous areas has been a major concern [1, 8], which highlights the further need for health-related interventions.

Sedentary behaviors are well-known risk factors of non-communicable diseases and are associated with high cardiovascular-related mortalities in prospective longitudinal studies [9]. In fact, sedentary behaviors are independent risk factors for chronic cardiovascular disease, meaning that being “sedentary” is associated with the prevalence of numerous diseases, even among those who do enough exercise [10–12].

More importantly, screen time dramatically increased during the COVID-19 pandemic. In this regard, several studies warned about the health consequences of this issue and warranted the need for health programs like encouraging regular physical activity, promoting educational opportunities, and increasing social support during the COVID-19 pandemic [13–16].

Among all the sedentary behaviors, screen-based sitting activities (such as using a computer, watching TV, and playing video games) need specific interventions because they are major contributors to overall sedentary time and are highly prevalent among children and adolescents [17–21]. The American Academy of Pediatrics (AAP) recommended reducing the daily screen time of children and teenagers to less than two hours, with no screen time for children under the age of two and less than one hour per day for children aged two to five [22, 23].

Screen-related behaviors are associated with a high prevalence of HTN, particularly among boys [17, 24]. The underlying mechanisms include increased arteriolar narrowing [25], obesity and poor sleep quality related to excessive screen use [24], increased stress, amygdala activation, as well as altering both sympathetic efferent output and responsiveness of hypothalamic–pituitary–adrenal axis [26, 27].

Several studies have been conducted on the correlation between HTN and screen time in children and adolescents. However, the achieved results regarding the role of screen type, gender, age, and geographic location are highly inconsistent. There is no summarized analysis of the quantity and quality of the correlation between screen time and HTN in children. Accordingly, this systematic review study was conducted to evaluate the relationship between screen time and HTN in children and adolescents. Also, in a dose–response meta-analysis, we further investigated the role of different factors such as screen type, age group, gender, time, and geographical distributions.

## Materials and methods

The results of this systematic review and meta-analysis were reported using the Preferred Reporting Items for Systematic Reviews and Meta‐Analyses (PRISMA) (Additional file 1: Table S1) [28]. The PRISMA 2020 statement includes a checklist to guide reporting of systematic reviews and consists of a 27-item checklist and a four-phase flow diagram. These items have been adapted for use by researchers conducting systematic reviews. Also, the abstract was developed using the PRISMA extension's comprehensive 12-item checklist. [29].

**Table 1:** The characteristics of included studies in the meta-analysis

References	Journal/Year	First author/Country	Setting/num	Design	Study group	Age (y)	HTN definition	BP measurement	ST definition	ST measurement	Main findings
[42]	Plos One/2020	Solomon-Moore E/UK	School/1283 + 797	Cross-sectional Prospective (OR reported)	Children	9–11	≤ 95th for age, sex and height	Omron 907	Video games/PC/TV/movies	Accelerometer	No correlation between sedentary time and HTN was reported. There was a significant positive relationship between sedentary time and high SBP
[40]	Scand J Public Health	Pederson J/Denmark	Community/964 + 963	Cross-sectional	Children	3–5	≤ 95th for age, sex and height	Electronic oscillometric	Video games/PC/tablet/TV/movies	Parent reported-daytime ST	No association between sedentary time and HTN was reported. A positive association between pre-bedtime ST and HTN was reported [1.57 (95% CI 1.02; 2.42) and 1.82 (95% CI 1.18; 2.89)], respectively, for 2–5 days/week and more than 6 times/week
[24]	BMC Pediatrics/2019	Zou Y/China	School/3737	Cross-sectional	Adolescents	12–15	≤ 95th for age, sex and height	Mercury Sphygmomanometer	Smartphone addiction	Smartphone Addiction Scale short version	Smartphone addiction was positively associated with HTN (OR = 2.205, 95% CI: 1.273–3.820)
[6]	Cien Saude Colet/2018	de Oliveira/Brazil	School/2524 + 3773	Cross-sectional	Adolescents	14–17	≤ 95th for age, sex and height	Omron HEM 742	Video games/PC/tablet/TV/movies	Self-reported	TV viewing was associated with high BP among boys. No significant association between ST and HTN among girls was reported
[4]	J Am Soc Hypert/2018	Karatazi K/Greece	School/1243 + 1230	Cross-sectional	Children + adolescents	9–13	≤ 95th for age, sex and height	Mercury Sphygmomanometer	Video games/PC/tablet/TV/movies	Self-reported	Boys in isolated systolic HTN had higher ST compared with others (*P* = 0.002); also, higher ST was associated with significantly higher odds of ISH (1.13 (1.04–1.23). No significant association between ST and HTN was observed
[17]	BMC Pediatrics/2018	Barstad LH/Norway	Clinic/268	Cross-sectional	Adolescents	12–18	≤ 95th for age, sex and height	Digital oscillimetric device, Dinamap ProCare	Time in front of the TV- or PC	Self-reported	Those in high ST group had higher SBP
[43]	Biomed Res Int/2017	WyszyNsk J/Poland	Community/568	Cross-sectional	Children + adolescents	7–18	≤ 95th for age, sex and height	Mercury Sphygmomanometer	Video games/PC/TV/movies	Self-reported	More than 2 h/d ST in school days was associated with higher odds of HTN [2.74 (1.25–6.04)]
[44]	Nutrients/2017	Gui ZH/China	Community/79,725	Cross-sectional	Children + adolescents	6- 17	≤ 95th for age, sex and height	Mercury Sphygmomanometer	Video games/PC/TV/movies	Self-reported	Those with more than 2h/d ST had higher odds of pre-HTN and HTN (5% and 6% higher risk, respectively)
[45]	Int J Obes/2017	Cureau FV/Brazil	School/36,956	Cross-sectional	Children + adolescents	12–17	≤ 95th for age, sex and height	Omron HEM 705	Video games/PC/TV	Self-reported	Those with more than 6 h/d ST had higher odds of HTN [1.21 (1.08–1.35); *P* = 0.003]
[46]	Iran J Public Health/2015	Safiri S/Iran	School/5625	Cross-sectional	Children + adolescents	10–18	≤ 95th for age, sex and height	Mercury Sphygmomanometer	TV/VCDs/PC	Self-reported	High SBP and DBP in those with high ST (*P* < 0.001)
[20]	Blood Pressure/2015	Christofaro DGD/Brazil	School/1231	Cross-sectional	Children + adolescents	10–18	≤ 95th for age, sex and height	Mercury Sphygmomanometer	TV/VCDs/PC	Self-reported	Higher odds of HTN (1.68) and higher ST in hypertensive compared with normotensive children and adolescents
[19]	Int J Obes/2014	NE Berentzen/Netherland	School/2651	Cross-sectional	Children + adolescents	11–12	≤ 95th for age, sex and height	Omron M6	TV/PC	Self-reported	No significant difference in SBP or DBP in highest versus lowest ST quartiles
[47]	Am J Prevent Med/2013	Stamatakis E/Portugal	School/2515	Cross-sectional	Children + adolescents	2–12	≤ 95th for age, sex and height	Omron M7	TV/VCDs/PC	Parent -reported	High SBP and DBP in those with high than 2 h/d screen behaviors
[18]	Psychosom Med/2013	Berendes A/Germany	Community/825	Cross-sectional	Children + adolescents	11–17	≤ 95th for age, sex and height	Sphygmomanometer	TV/VCDs/PC	Self-reported	Higher odds of HTN among those with more than 2 h/d TV, VCD and more than 0.5 h/d PC exposure (*P* < 0.001)
[48]	J Korean Med Sci/2012	Byun W/Korea	Community/577	Cross-sectional	Children + adolescents	12 -18	≤ 95th for age, sex and height	Sphygmomanometer	TV/VCDs/PC	Self-reported	No significant association between odds of HTN and screen behaviors
[41]	BMC Public Health/2011	Carson V/USA	Community/2527	Cross-sectional	Children + adolescents	6–19	Highest versus lowest BP quartiles	Sphygmomanometer	TV/PC	Accelerometer	No significant association between odds of HTN and screen behaviors
[49]	J Sports Sci/2010	Ullrich-French SC [49]/USA	School/153	Cross-sectional	Adolescents	11–15	≤ 95th for age, sex and height	Sphygmomanometer	TV/VCDs/PC	Self-reported	Higher SBP in those with more than 2 h/d screen behaviors (*P* < 0.001)
[50]	Arch Ped Adol Med/2010	Hardy LL/Australia	School/496	Cross-sectional	Adolescents	14–17	≤ 95th for age, sex and height	Mercury Sphygmomanometer	TV/DVDs/videos/PC for recreation	Self-reported	Odds of higher DBP in boys with more than 2 h/d screen behaviors [3.30 (1.35–8.12; *P* < 0.001)]
[51]	J Hum Hyper/2009	Lazarou C/Cyprus	School/622	Cross-sectional	Adolescents	10–13	≤ 95th for age, sex and height	Mercury Sphygmomanometer	TV	Self-reported	No significant association between odds of HTN and TV watching
[26]	Am J Prev Med/2007	Pardee PE/USA	Clinic/546	Cross-sectional	Children + adolescents	4–17	≤ 95th for age, sex and height	Self-reported	TV	Parent and self-reported	Higher odds of HTN among those with 2–4 h/d and more than 4 h/d compared with those with less than 2 h/d TV watching [OR:2.54 (1.51–4.29 and OR:3.29 (1.95–5.59, respectively]

*HTN* hypertension, *TV* television, *ST* screen time, *BP* blood pressure, *SBP* systolic blood pressure, *DBP* diastolic blood pressure, *PC* personal computer, *DVD* digital video discs, *VCDs* video compact disc digital

All of the studies evaluated both genders. Except of the study by Pardee [26] that was performed among obese children and adolescents, other studies recruited apparently healthy children and adolescents

### Search strategy

In this study, three electronic databases, including Scopus, PubMed, and Embase, were searched systematically to find the existing analyses of the association between screen time and HTN up to 19 July 2022. There search was limited to English language articles. Also, we manually searched the reference lists of all the retrieved systematic reviews, papers, and meta-analyses for any publications that may have been overlooked.

Our search method for each of these electronic databases was based on a combination of PubMed's MeSH (Medical Subject Headings) phrases and free-text words. The PubMed search method was as follows: [(hypertension) OR (HTN) OR (systolic blood pressure) OR (diastolic blood pressure) OR (SBP) OR (DBP) OR (blood pressure) OR (hypertension) AND (child) OR (children) OR (teen) OR (adolescent) OR (boy) OR (girl) OR) OR (pediatric) OR (youth) OR (teenager) OR (toddler) AND ((sedentary behavior) OR (screen time) OR (sitting time) OR (sitting time) OR (television view) OR (watching television) OR (computer use) OR (internet use) OR (smartphone) OR (video game) OR (electronic game) OR (depress) (Additional file 1: Table S2).

**Table 2:** Subgroup analysis for the association between screen time and hypertension among children and adolescents

Group	No. of studies*	OR (95% CI)			*P*_within group_	*P*_between group *_	*P*_heterogeneity_	*I*^2^ (%)
Total	15 (with 44 individual studies)	1.153	1.076	1.234	< 0.001		< 0.001	83.2
*Continent*						< 0.001	< 0.001	
America	9	1.325	1.015	1.730	0.038		< 0.001	72.2
Europe	25	1.055	1.018	1.093	0.003		< 0.001	59.1
Asia	5	1.034	0.951	1.124	0.432		0.024	64.6
Australia	5	1.460	0.947	2.252	0.087		0.229	28.9
*Screen type*						< 0.001		
TV	6	1.333	0.863	2.060	0.195		< 0.001	85.4
Video game, TV, PC	32	1.050	1.021	1.079	0.001		0.002	47.2
Video game, PC	1	0.770	0.344	1.722	0.524		–	0
TV, video game	1	1.470	1.225	1.763	< 0.001		–	0
PC, video game	1	1.000	0.874	1.145	0.99		–	0
CF	1	2.205	1.273	3.820	0.005		–	0
PC	2	1.233	0.723	2.100	0.442		0.144	53.1
*HTN type*						< 0.001		
ISH	9	1.026	0.990	1.063	0.164		0.269	19.6
IDH	7	1.028	0.989	1.070	0.160		0.124	40.1
Total HTN	28	1.169	1.086	1.258	< 0.001		< 0.001	70.1
*Gender*						< 0.001		
Boys	12	1.070	1.012	1.131	0.017		0.005	59.3
Girls	11	1.012	0.987	1.038	0.332		0.865	0.0
Both	21	1.189	1.084	1.303	< 0.001		< 0.001	73.4
*Age group*						< 0.001		
Children	17	1.038	1.009	1.067	0.011		0.017	47.1
Adolescents	10	1.262	0.951	1.674	0.107		0.052	46.4
Both	17	1.148	1.046	1.260	0.004		< 0.001	74.8
*Setting*						< 0.001		
Community	10	1.142	1.031	1.263	< 0.001		< 0.001	73.7
School	33	1.053	1.016	1.090	< 0.001		< 0.001	55.2
Clinic-based	1	3.290	1.948	5.555	< 0.001		< 0.001	0
*ST measurement tool*						< 0.001		
Accelerometer	10	1.017	0.994	1.040	0.152		0.734	0.0
Questionnaire	34	1.076	1.037	1.117	< 0.001		< 0.001	67.9
*Sample size*						< 0.001		
1000>	21	1.063	0.978	1.156	0.148		< 0.001	60.1
1000–5000	20	1.091	1.039	1.147	0.001		< 0.001	69.4
≥ 5000	3	1.089	1.023	1.160	0.008		0.043	68.1
Study quality*						< 0.001		
Moderate	18	1.074	1.024	1.127	0.003		0.012	48.1
High	26	1.080	1.024	1.139	0.005		< 0.001	72.6

*TV* television, *PC* personal computer, *VG* video game, *CF* cell phone, *ISH* isolated systolic hypertension, *IDH* isolated diastolic hypertension, *ST* screen time

*Low quality = 0–3; moderate quality = 4–7; high quality ≥ 8

### Study selection

**Fig. 1:**
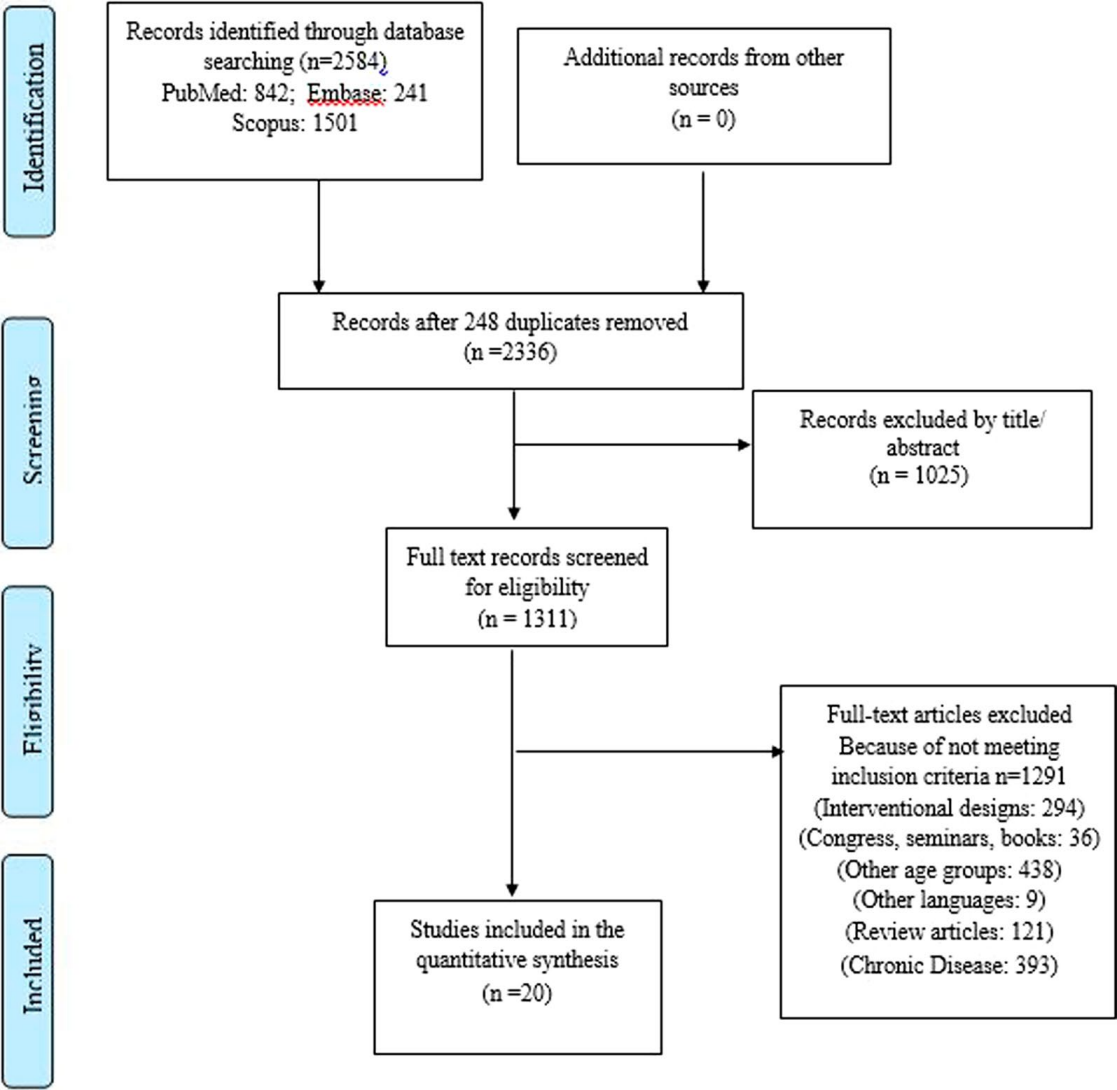
Study flowchart

Our search approach generated 2821 articles in total. We imported the remaining papers (*n* = 2519) into EndNote after deleting the duplicates. Two investigators checked all articles independently (SA, MAF). Then, out of 1396 manuscripts that remained for full-text screening, 1376 articles were rejected due to inappropriate subjects, different designs, involving other age groups and languages, being conference and seminar reviews, investigating other chronic diseases not related to metabolic abnormalities (like asthma, intellectual disabilities, depression), and evaluating different parameters. Any disagreements between reviewers were settled through dialogue. The final meta-synthesis included 20 manuscripts (Fig. 1).

## Inclusion and exclusion criteria

The following conditions were considered as inclusion criteria: (1) observational research (case–control, cross-sectional, or cohort studies with baseline assessment of study parameters); (2) studies examining the correlation between screen time and hypertension or blood pressure (isolated systolic or diastolic HTN or total HTN, systolic or diastolic blood pressure); (3) research conducted on children and adolescents aged 18 or older; and (4) studies presenting the odds ratio of the relationship between HTN and screen time or providing mean ± standard deviation (SD) of systolic blood pressure (SBP) and diastolic blood pressure (DBP). Only cross-sectional data from cohort studies (at baseline or after a period of follow-up) that reported odds ratio (OR) were considered. Studies that did not provide an OR or mean (SD) blood pressure values were also disqualified. These included clinical trials, systematic reviews, meta-analyses, case reports, case series, experiments, short communications, letters to the editor, and those that considered the associations other than the one between screen time and HTN.

### Data extraction and quality assessment of included studies

Two researchers independently extracted data using a normal Excel extraction datasheet. The following information was extracted from each article: first author, journal name, nation, publication year, age range of subjects, research design, final sample size and the number of participants in each category of screen time or blood pressure, adjusted covariate, gender, setting, blood pressure, screen time definition, blood pressure and screen time measurement tools, and major findings. Discrepancies between reviewers were resolved by discussion.

The Agency for Healthcare Research and Quality (AHRQ) checklist was used to evaluate the listed studies for their methodological quality [30]. The AHRQ guidance on evaluating the strength of evidence differentiates between the evaluation of precision and risk of bias. The types of bias that the AHRQ checklist evaluates include selection bias, performance bias, attrition bias, detection bias, and reporting bias [31]. For each item, one point was given for a "YES" response, and zero point was given for a "NO" or "UNCLEAR" response. Overall, the quality ranged from poor (a score of 0–3) to satisfactory (a score of 4–7) or excellent (a score of 8 and greater). In addition, the quality of studies was not considered for including or excluding the articles (Additional file 1: Table S3). We also evaluated the certainty of evidence using the Grading of Recommendations Assessment, Development, and Evaluation (GRADE) framework (Additional file 1: Table S4) [32, 33].

**Table 3:** Details of nonlinear association between screen time and hypertension among children and adolescents

Variable	ST increment (min/day)	OR for HTN	*P*	95% CI
Children	1	0.003	**0.012**	0.001, 0.006
	50	1.178		1.036, − 1.340
	100	1.389		1.073, 1.797
	200	1.929		1.1523, 3.229
Adolescents	1	0.002	0.074	− 0.001, 0.003
	50	1.082		0.992, 1.180
	100	1.171		0.984, 1.393
	200	1.372		0.970, 1.940
Children + Adolescents	1	0.0027	**0.008**	0.001, 0.004
	50	1.146		1.036, 1.270
	100	1.314		1.073, 1.610
	200	1.727		1.152, 2.590
TV, video games, PC	1	0.001	0.140	− 0.001, 0.002
	50	1.049		0.984, 1.120
	100	1.102		0.968, 1.252
	200	1.214		0.938, 1.56
TV	1	0.002	**0.001**	0.001, 0.040
	50	1.131		1.0498, 1.219
	100	1.280		1.102, 1.486
	200	1.637		1.214, 2.207

**Abbreviations**: HTN, hypertension; TV, television; PC, personal computer; ST, screen time; CI, confidence interval; Bold values represent the significant threshold of less than <0.05

### Statistical analysis

We utilized STATA version 13 (STATA Corp, College Station, TX, USA) to analyze data. Also, *p* values less than 0.05 were regarded as statistically significant.

### Statistical analysis of two-class meta-analysis

Two-class meta-analysis is the comparison of an outcome variable between two study groups regardless of the nature of variable. The present study identified three approaches to two-class meta-analysis as a bivariate analysis: first, the studies examining the relationship between odds of total or isolated HTN and screen time; second, the investigations reporting the comparison between SBP or DBP [mean (SD)] in those with the highest versus lowest screen times; and third, the studies evaluating the comparison of screen time [mean (SD)] in hypertensive versus normotensive children and adolescents. Therefore, the OR and 95% confidence interval (CI) or the mean and SD of the variable were used to determine the unstandardized effect size calculated by the pooled estimate of the OR or the weighted mean difference (WMD) with 95% CI.

When ORs were not provided but information about exposure to the variable across groups was available, we used the prevalence odds ratios (PORs) proposed by Pearce N as the most accurate way to gauge the magnitude of an effect [34] as follows: $\mathrm{POR}=\left[\frac{P1}{1-P1}\right]/\left[\frac{P0}{1-P0}\right]$. In this formula, *P*0 and *P*1 represent the prevalence in exposed and non-exposed groups, respectively. The approach proposed by Hozo et al. [35] was utilized when the median and range were reported rather than the mean and SD. In this method, the median values are regarded to be the best approximation of the mean when the sample size is greater than 25 and the SD is determined as follows: ${S}^{2}\approx (\frac{1}{12}(\frac{{\left(a-2m+b\right)}^{2}}{4}+{\left(b-a\right)}^{2}$) [35]; while *m* is median, *a* and *b* are low and high end of the ranges, respectively.

The method proposed by Walter and Yao as an upgraded version of the "range" method was applied for missing SDs. In this method, SD is calculated as follows: SD = (*b*−*a*)/4 [36, 37], where *a* and *b* are low and high end of the ranges, respectively. We assumed that each category would have an equal number of participants if the number of participants in each category was not provided.

All the screen time data were converted to min/day for dose–response meta-analysis or hours/day for two-class meta-analysis. Cochran's *Q* and *I*^2^ tests were utilized to identify the heterogeneity between the studies as follows: *I*^2^ ˂ 25%, no heterogeneity; *I*^2^ = 25–50%, moderate heterogeneity; and *I*^2^ > 50%, high heterogeneity [38]. The heterogeneity was considered significant if either the *Q* statistic had *P* < 0.1 or *I*^2^ > 50%.

As suggested by Riley et al*.* [39], the random-effects model was used due to the high heterogeneity values. We used subgrouping to identify the possible sources of heterogeneity based on the continent, screen type, HTN type, gender, age group, screen time measurement tool, setting, sample size, and quality of the study. Publication bias was evaluated using the Begg's funnel plots, Begg's adjusted rank correlation, and Egger's regression asymmetry tests.

#### Statistical analysis of dose–response meta-analysis

The dose–response meta-analysis included studies that examined at least three categories for screen time and the odds or prevalence of HTN as a multivariate analysis. Accordingly, nine individual studies in five articles were included [6, 24, 26, 40, 41]. If possible, the isolate dose–response meta-analysis was applied to the variables included in at least three individual studies. For example, a separate dose–response analysis was applied for the studies performed among children, adolescents, or a combination of both, as well as for the studies that evaluated watching TV, using personal computers (PC), and playing video games (VG).

The median point for each category of screen time was determined. If medians were not supplied, they were calculated using the midpoint of the minimum and maximum values. When the minimum or maximum screen time categories were undefined, the screen time was estimated by assuming a similar interval and calculating the midpoint. Assuming ORs and CIs of 1, the lowest category was the reference category.

By creating limited cubic splines with three knots at defined percentiles (10%, 50%, and 90%) of distribution, we were able to examine the potential nonlinear relationships using random-effects dose–response meta-analysis and derive study-specific ORs, which were reported as ORs of HTN for every 1, 50, 100, and 200 min increment in screen time.

## Results

### Characteristics of included studies

Table 1 provides the details of the evaluated studies. We included 20 studies in the two-class meta-analysis with a total number of 151,763 participants [4, 6, 17–20, 24, 26, 40–51]. Several studies reported the results in different subgroups of HTN, isolated SBP or DBP, genders, or age groups. The results were therefore isolated as individual studies.

Solomon-Moore et al*.* [42] evaluated the correlation between inactivity and blood pressure in primary school children. This study comprised eight subgroups that individually evaluated isolated systolic and diastolic HTN at ages nine and 11 in both girls and boys. The researchers reported a positive correlation between inactivity and high isolated systolic HTN among girls [OR: 1.08 (1.01 to 1.16)].

Pedersen et al*.* [40], in a cross-sectional study of Odense Child Cohort study, reported the relationship between parent-reported screen time either in daytime or before bedtime with hypertension in children aged five; prolonged exposure to screen before bedtime (two to five days/week and more than six days/week) was positively correlated with high blood pressure [OR: 1.57 (95% CI: 1.02–2.42) and OR: 1.82 (95% CI: 1.18–2.89), respectively].

Oliveira et al*.* [6] analyzed the correlation between several components of screen time, such as watching television, playing video games, and/or using the PC for less than two hours, two to four hours, and more than four hours in both genders. So, a total of four studies demonstrating a positive correlation between TV television and HTN in boys, but not girls, were included in the analysis.

Karatzi et al*.* [4] investigated the correlation between screen time and isolated systolic hypertension (ISH), isolated diastolic hypertension (IDH), and total HTN separately for boys and girls; therefore, it was included as six isolate studies in the meta-analysis. According to the results, only boys with higher ISH had higher screen time (*P* = 0.002). Also, a higher screen time was correlated with significantly higher odds of ISH [OR: 1.13 (CI: 1.04–1.23)]. Meanwhile, no association among girls was reported.

Wyszyńska et al*.* [43] analyzed the correlation between screen time and HTN in intellectually disabled children compared with the control group. So, we only included the results of healthy children and adolescents. The results were included as six individual studies analyzing the correlation between screen time and odds of HTN in school days and weekends in normotensive, pre-hypertensive, and hypertensive children and adolescents. According to the results, screen time of more than 2 h/day on school days was correlated with higher odds of HTN [2.74 (1.25–6.04)].

Two separate studies demonstrating a relationship between screen time and the risk of pre-HTN and HTN, such as that conducted by Gui et al. [43], were included in the meta-analysis. In this study, subjects with a screen time of more than 2 h/day had 5% and 6% higher risks of pre-HTN and HTN, respectively.

The study by Berendes et al*.* [18] evaluated the correlation between HTN and TV/video games or PC separately and reported higher odds of HTN in people with high screen-related behaviors. Similarly, Byun et al*.* [48] conducted two individual studies on the association between HTN and TV or PC/video games and reported higher odds of HTN among those with more than 2 h/day TV and video CD activity and more than 0.5 h/day PC exposure (*P* < 0.001). Carson et al*.* [41] reported the association between TV or PC and HTN, which was included as two studies. The authors reported no significant relationship between the odds of HTN and screen behaviors.

Hardy et al*.* [50] investigated the odds of ISH, IDH, and total HTN in adjusted and unadjusted models for boys and girls. So, five individual data sets were extracted to be included in the two-class meta-analysis; a significant association was only reported for the OR of higher DBP in boys with more than 2 h/day screen-related behaviors [OR: 3.30; CI: 1.35–8.12; *P* < 0.001)].

Five other studies [20, 24, 26, 45, 51] were included as single studies; for example, the study by Zou et al*.* [24], which reported a significant correlation between smartphone addiction and HTN among junior school students in China [OR: 2.205, 95% CI: 1.273–3.820].

### The results of the two-class meta-analysis

**Fig. 2:**
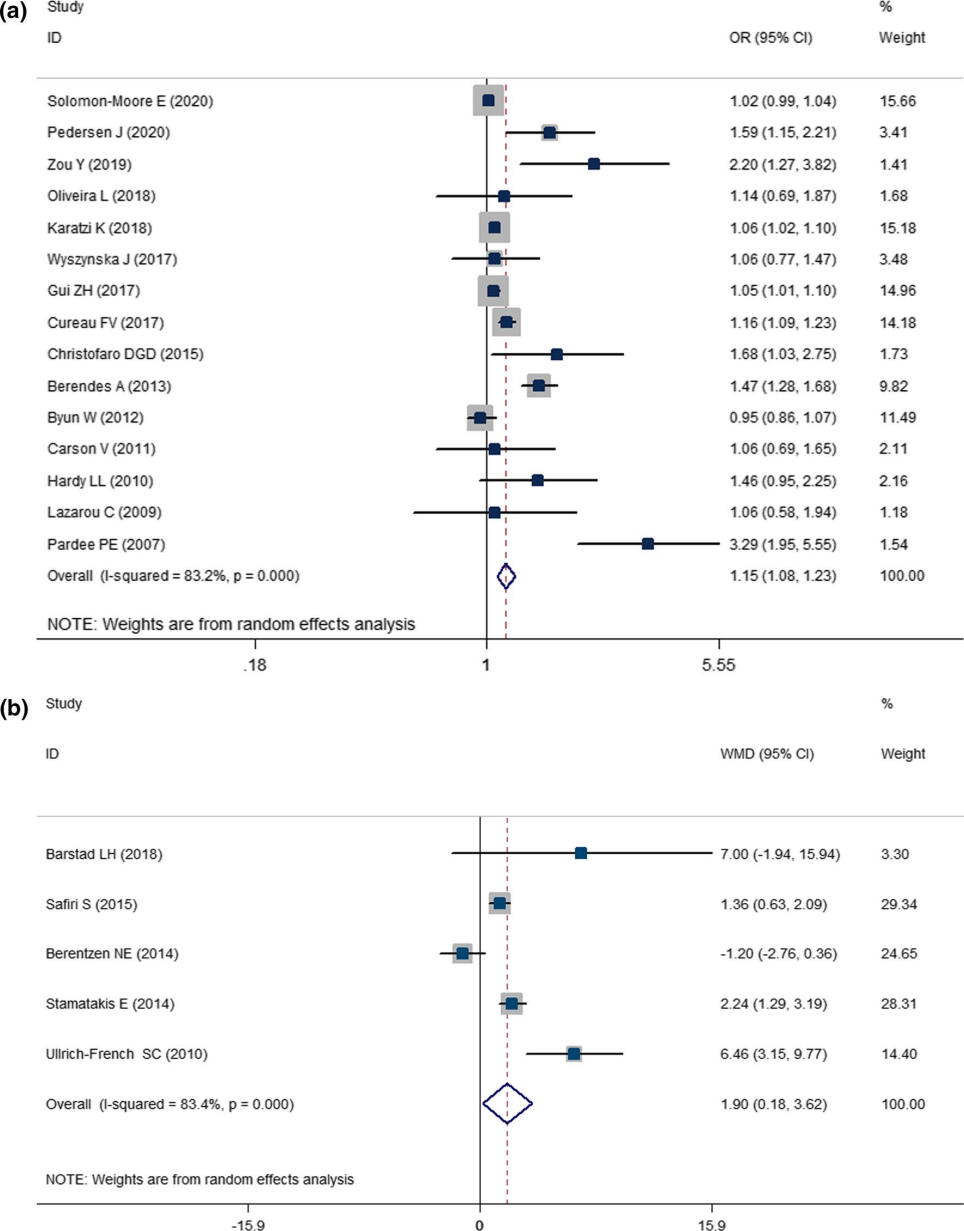

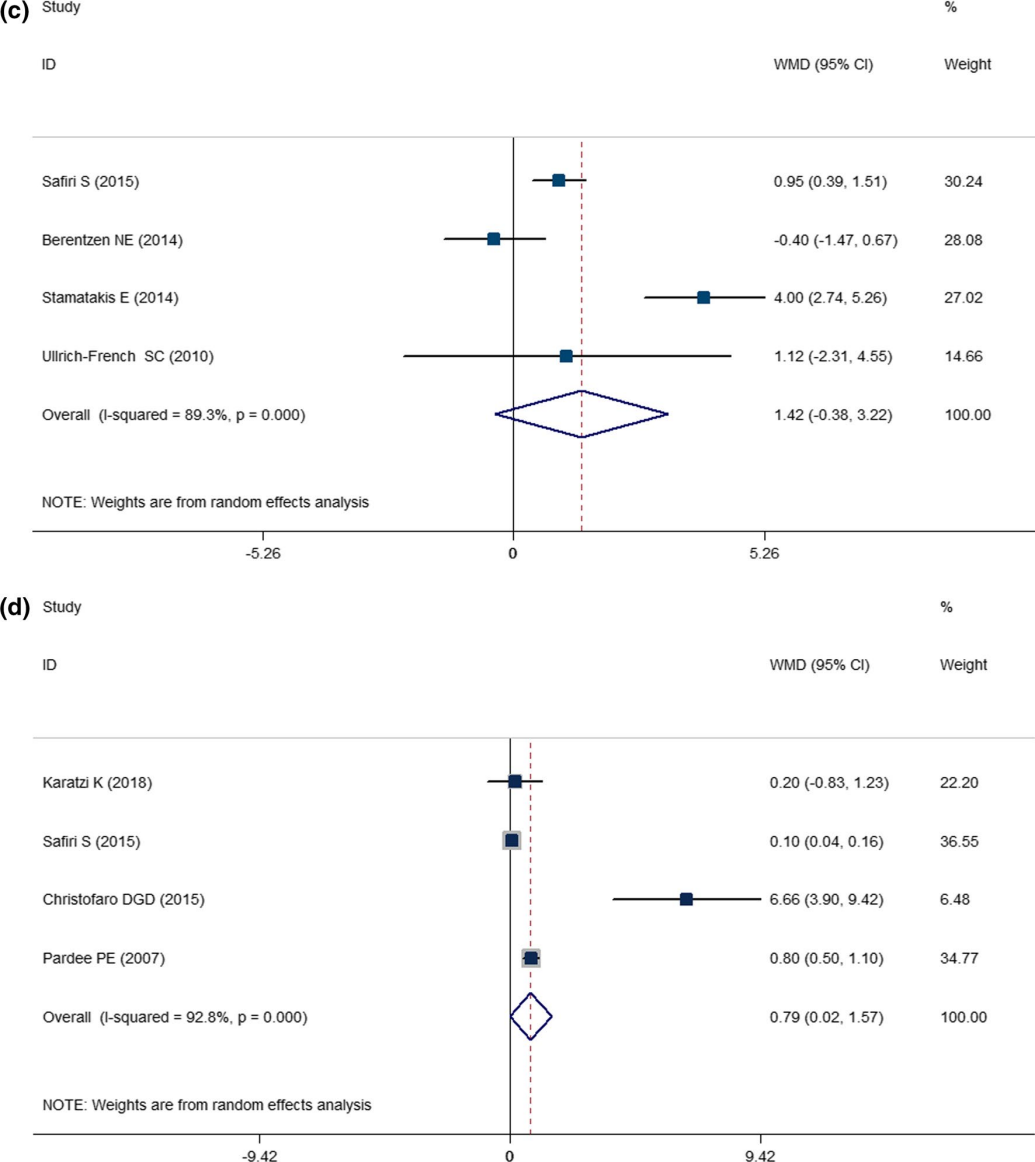
Association between screen time and **A** pooled odds ratio (OR) of hypertension; **B** weighted mean difference (WMD) with 95% confidence interval (CI) of SBP, **C** weighted mean difference (WMD) with 95% confidence interval (CI) of DBP, and **D** weighted mean difference (WMD) with 95% confidence interval (CI) for the screen time in hypertensive versus normotensive youth. *I*^2^ represents the degree of heterogeneity

The present two-class meta-analysis demonstrated the correlation between OR of HTN and screen time (Fig. 2A). High levels of screen time were correlated with an increased risk of HTN by about 15% (OR: 1.153; CI: 1.076, 1.234; *P* < 0.001; *I*^2^ = 83.2%).

The comparison of SBP and DBP between the highest and lowest categories of screen time using a two-class meta-analysis is presented in Fig. 2B, C. As can be seen, the maximum level of screen time was associated with a 1.9 mmHg increase in SBP (WMD: 1.898; CI: 0.181, 3.616; *P* = 0.030; *I*^2^ = 83.4); however, 1.42 mmHg increase in DBP in the highest versus lowest screen time categories was not statistically significant (WMD = 1.420; CI = − 0.383, 3.223; *P* = 0.123; *I*^2^ = 89.3).

Figure 2D presents the results of the two-class meta-analysis of the comparison of screen time in hypertensive versus normotensive children and adolescents. As can be seen, hypertensive children and adolescents had 0.79 h (47.4 min) higher screen time compared with normotensive children and adolescents (WMD: 0.791; CI: 0.015, 1.566; *P* = 0.046; *I*^2^ = 92.8).

Subgrouping was performed to find the source of heterogeneity (Table 2). Subgroupings according to the continent, HTN type, or age group were accompanied by a slight reduction in heterogeneity; however, there was no heterogeneity in the case of girls, as well as the studies that used an accelerometer to measure screen time. Also, in subgrouping according to sample size, there was a reduction in heterogeneity values in all the subgroups (e.g., 1000>, 1000–5000, and 5000≤).

### The results of dose–response meta-analysis

**Fig. 3:**
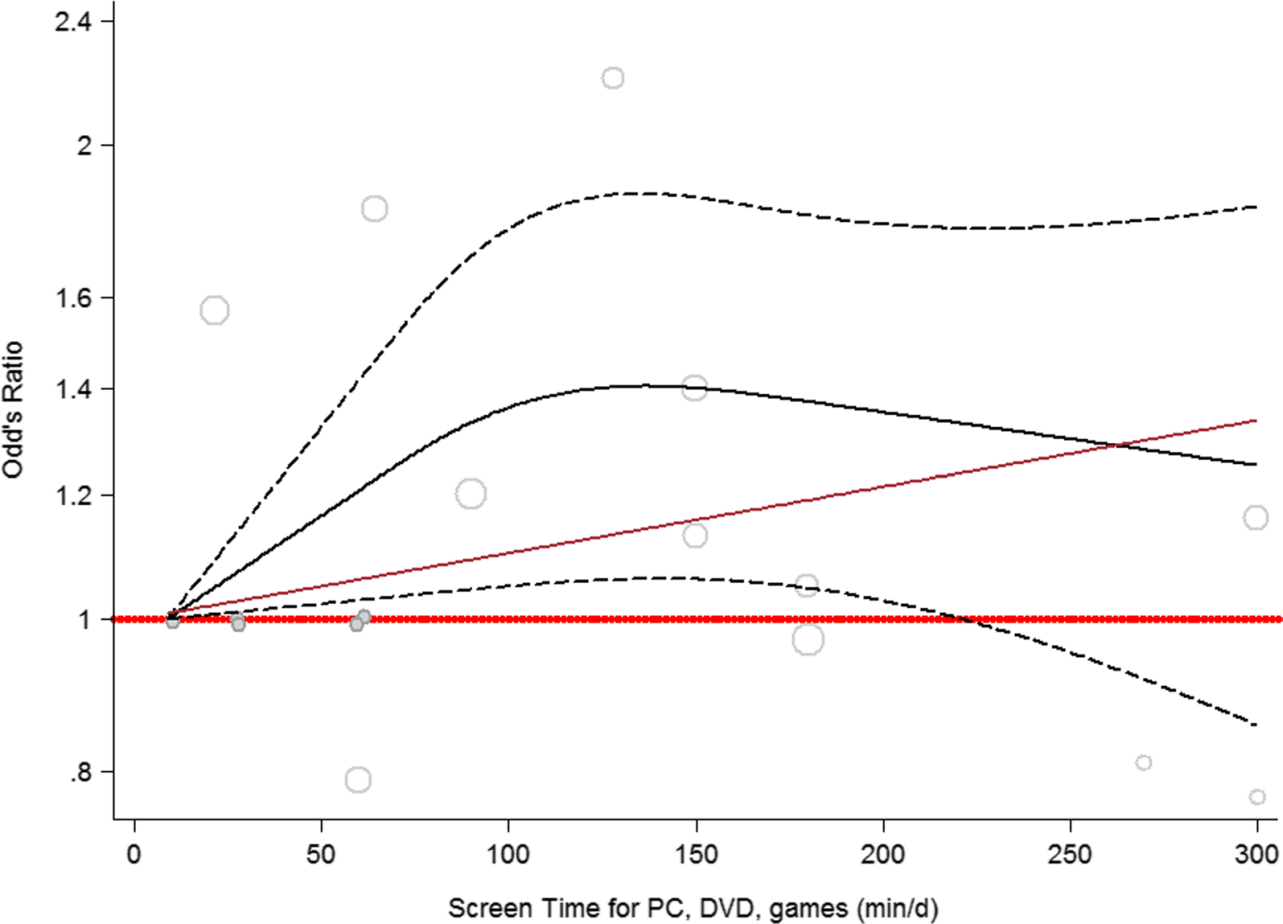
Dose–response association between screen time and odds of hypertension. Linear relation (solid line) and 95% CI (dashed lines) of pooled OR of HTN prevalence by 1 min/day increment of screen time of DVD, PC, and video games (*P*_nonlinearity_ = 0.0636) among children and adolescents

Figure 3 displays the dose–response relationship between screen time and hypertension, indicating a nonlinear correlation between longer periods of watching TV or playing video games and the development of hypertension (*P*_nonlinearity_ = 0.049). However, there was no deviation from linearity in the link between HTN and screen time among teenagers (Additional file 1: Figure S1A), children + adolescents (Additional file 1: Figure S1B), and TV (Additional file 1: Figure S1C). A separate analysis for children was not possible because the number of studies was not sufficient for figuring at least three cubic display-knots. The dose–response associations for all subgroupings were statistically significant except for those studies that assessed only TV as the screen type (*P* < 0.05).

Table 3 depicts the details of the dose–response association. It can be inferred that by 50-, 100-, and 150-min increments in screen time, there is 17%, 38%, and 92% increased risk of hypertension among children, 8%, 17%, and 32% in teenagers, and 14%, 31%, and 72% in the combination of children and adolescents. Accordingly, a 4%, 10%, and 21% increase in the odds of HTN was observed with 50-, 100-, and 150-min increases in screen time of TV, video games, and PC; these increments for TV were 13%, 27%, and 63%, respectively.

### The results of quality assessment and publication bias

According to the quality evaluation results based on the AHRQ checklist (Additional file 1: Table S3), while 18 individual studies had average quality scores, 26 studies had very high quality. Publication bias was assessed using the OR funnel plot for HTN (Additional file 1: Figure S1). Additionally, the publication bias was clarified using Begg's and Egger's regression tests.

We found no evidence of publication bias for any of the measured variables [OR of HTN and screen time: Egger’s test (*P* = 0.811) and Begg’s test (*P* = 0.800); SBP in highest versus lowest screen time category: Egger’s test (*P* = 0.600) and Begg’s test (*P* = 0.624); DBP in highest versus lowest screen time category: Egger’s test (*P* = 0.771) and Begg’s test (*P* = 0.497); and screen time in hypertensive versus normotensive children and adolescents: Egger’s test (*P* = 0.072) and Begg’s test (*P* = 0.435)].

## Discussion

This systematic review and meta-analysis evaluated the findings of studies examining the association between screen time and blood pressure in children and adolescents. The results revealed that high screen time increased the odds of HTN by 7% and SBP by 1.898 mmHg. This warrants the need for educational or therapeutic interventional programs to overcome this health issue among children and adolescents.

Increased blood pressure among children due to sedentary behaviors is associated with increased brachio-ankle pulse wave velocity and intima-media thickness [52, 53]. Although the correlation between HTN and screen time was attributed to obesity and overweight in several studies [54, 55], in some other studies, the relationship between screen time and HTN was independent of body weight status. While Tebar et al*.* [56] reported that the association between HTN and sedentary behavior was more pronounced among normal-weight rather than obese children, Wennberg et al*.* [57] stated that TV watching was associated with HTN independent of BMI status [57]. Several other studies also reported similar results [58, 59].

In our subgrouping according to gender, the correlation between HTN and screen time was only significant among boys or the combination of both genders, but not among girls. Generally, boys have a higher tendency toward screen-related behaviors compared with girls. This was more pronounced in engagement in video games, videocassette recorder (VCR) playing [60], and television watching [61]; also, boys were more engaged in long-lasting screen-related activities [62–64]. In addition, the prevalence of HTN varies based on gender; generally, boys have higher baseline SDP and DBP compared with girls because of higher stroke volume and higher total peripheral resistance during stressful situations [18, 65, 66].

In subgrouping the results according to age, the relationship between HTN and screen time was only significant among children, but not adolescents; it seems that children at lower ages are at greater risk of sleep disturbance and low sleep duration due to screen-related behaviors [67–69], and sleep disorder is one of the leading causes of childhood HTN [70, 71].

In subgrouping by continent, the results for studies done in Europe and the United States were statistically significant, whereas the results for other groups were insignificant. This may be explained by the greater volume of investigations conducted on these two continents.

The nonlinear association was observed in the correlation between high screen time for DVD, PC, and video games and HTN (*P*_nonlinearity_ = 0.049). This nonlinear association revealed the highest odds of HTN (~1.4) in 100–150-min/day screen time of DVDs, PC, and video games, which is compatible with the recommendations of the AAP on reducing the daily screen time of children and teenagers to less than two hours [22].

The current study had some limitations. First, we only included observational studies in the meta-analysis, which makes causal inference impossible. Second, there was no study evaluating the separate effects of video games. So, the pure effect of video games as a screen type on blood pressure could not be extracted.

In conclusion, the findings of this review can be helpful because of the growing tendency to screen-related behaviors during the COVID-19 pandemic, which limited the usual physical activities of children and adolescents outdoors and made them follow their school lessons online. So, policymakers should be aware of the future health consequences of limited physical activity, even at young ages, and design programs to substitute children’s screen-addictive behaviors with regular physical activities either at home or outside. Another critical issue is the high prevalence of overeating behaviors and higher intakes of junk and fast foods during screen watching, which is a strong promoter of obesity, metabolic syndrome, and HTN independently of body weight status [72–77]. Therefore, educating families to improve dietary behaviors and reduce “empty calorie” eating is essential.

### Supplementary Information

**Additional file 1.** Supplementary Tables and Figures.

## Data Availability

The data are available upon reasonable request from the corresponding author.
