# Supplementary material for: Prolonged screen watching behavior is associated with high blood pressure among children and adolescents: a systematic review and dose–response meta-analysis
Source: J Health Popul Nutr. 2023 Aug 31;42:89. doi: 10.1186/s41043-023-00437-8 (PMC10468885; doi:10.1186/s41043-023-00437-8)
Supplement: Supplementary file 1 — Additional file 1. Supplementary Tables and Figures. [file 41043_2023_437_MOESM1_ESM.docx]

**Title: Prolonged screen watching behavior is associated with high blood pressure among children and adolescents: A systematic review and dose-response meta-analysis**

**Supplementary Material**

**Sup. Table 1.** PRISMA Checklist ^1^

| **Section/topic** | **#** | | | **Checklist item** | **Reported on page #** |
| --- | --- | --- | --- | --- | --- |
| **TITLE** | | | | |  |
| Title | 1 | | | Identify the report as a systematic review, meta-analysis, or both. | Page 1; line 1-3 |
| **ABSTRACT** | | | | |  |
| Structured summary | 2 | | | Provide a structured summary including, as applicable: background; objectives; data sources; study eligibility criteria, participants, and interventions; study appraisal and synthesis methods; results; limitations; conclusions and implications of key findings; systematic review registration number. | Page 2; lines 1-21 |
| **INTRODUCTION** | | | | |  |
| Rationale | 3 | | | Describe the rationale for the review in the context of what is already known. | Page 3 lines 2-13 |
| Objectives | 4 | | | Provide an explicit statement of questions being addressed with reference to participants, interventions, comparisons, outcomes, and study design (PICOS). | Page 4 lines 1-2 |
| **METHODS** | | | | |  |
| Protocol and registration | 5 | Indicate if a review protocol exists, if and where it can be accessed (e.g., Web address), and, if available, provide registration information including registration number. | | | Page 4 lines 4-8 |
| Eligibility criteria | 6 | Specify study characteristics (e.g., PICOS, length of follow-up) and report characteristics (e.g., years considered, language, publication status) used as criteria for eligibility, giving rationale. | | | Page 4; lines 23-28  Page 5; line 1-16 |
| Information sources | 7 | Describe all information sources (e.g., databases with dates of coverage, contact with study authors to identify additional studies) in the search and date last searched. | | | Page 4, line 10-21 |
| Search | 8 | Present full electronic search strategy for at least one database, including any limits used, such that it could be repeated. | | | Page 4, line 10-21 |
| Study selection | 9 | State the process for selecting studies (i.e., screening, eligibility, included in systematic review, and, if applicable, included in the meta-analysis). | | | Page 4; lines 23-28  Page 5; line 1-16 |
| Data collection process | 10 | Describe method of data extraction from reports (e.g., piloted forms, independently, in duplicate) and any processes for obtaining and confirming data from investigators. | | | Page 5; lines 17-28 |
| Data items | 11 | List and define all variables for which data were sought (e.g., PICOS, funding sources) and any assumptions and simplifications made. | | | Page 5; lines 17-26  Page 6; lines 1-4 |
| Risk of bias in individual studies | 12 | Describe methods used for assessing risk of bias of individual studies (including specification of whether this was done at the study or outcome level), and how this information is to be used in any data synthesis. | | | Page 5; lines 17-28 |
| Summary measures | 13 | State the principal summary measures (e.g., risk ratio, difference in means). | | | Page 6; lines 1-27  Page 7; lines 1-20 |
| Synthesis of results | 14 | Describe the methods of handling data and combining results of studies, if done, including measures of consistency (e.g., I^2^) for each meta-analysis. | | | Page 6; lines 1-27  Pahe 7; lines 1-20 |
| Risk of bias across studies | 15 | | Specify any assessment of risk of bias that may affect the cumulative evidence (e.g., publication bias, selective reporting within studies). | | Page 6; lines 1-27  Page 7; lines 1-27 |
| Additional analyses | 16 | | Describe methods of additional analyses (e.g., sensitivity or subgroup analyses, meta-regression), if done, indicating which were pre-specified. | | Page 6; lines 1-27  Page 7; lines 1-20 |
| **RESULTS** | | | | |  |
| Study selection | 17 | | Give numbers of studies screened, assessed for eligibility, and included in the review, with reasons for exclusions at each stage, ideally with a flow diagram. | | Figure 1 |
| Study characteristics | 18 | | For each study, present characteristics for which data were extracted (e.g., study size, PICOS, follow-up period) and provide the citations. | | Page 7; lines 22-28  Page 8  Page 9; lines 1-11 |
| Risk of bias within studies | 19 | | Present data on risk of bias of each study and, if available, any outcome level assessment (see item 12). | | Page 10; lines 18-27 |
| Results of individual studies | 20 | | For all outcomes considered (benefits or harms), present, for each study: (a) simple summary data for each intervention group (b) effect estimates and confidence intervals, ideally with a forest plot. | | Figure 2A, B, C,D |
| Synthesis of results | 21 | | Present results of each meta-analysis done, including confidence intervals and measures of consistency. | | Figure 2A, B, C, D |
| Risk of bias across studies | 22 | | Present results of any assessment of risk of bias across studies (see Item 15). | | Page 10; lines 18-27 |
| Additional analysis | 23 | | Give results of additional analyses, if done (e.g., sensitivity or subgroup analyses, meta-regression [see Item 16]). | | Figure 3A, B, C, D |
| **DISCUSSION** | | | | |  |
| Summary of evidence | 24 | | Summarize the main findings including the strength of evidence for each main outcome; consider their relevance to key groups (e.g., healthcare providers, users, and policy makers). | | Page 11; lines 1-4 |
| Limitations | 25 | | Discuss limitations at study and outcome level (e.g., risk of bias), and at review-level (e.g., incomplete retrieval of identified research, reporting bias). | | Page 12 |
| Conclusions | 26 | | Provide a general interpretation of the results in the context of other evidence, and implications for future research. | | Page 12; lines 7-16 |
| **FUNDING** | | | | |  |
| Funding | 27 | | Describe sources of funding for the systematic review and other support (e.g., supply of data); role of funders for the systematic review. | | Page 13 |

**Sup. Table 2**. Search strategies and the number of records according to different electronic database

| **Search strategy** | **Database** | **Num. of records** |
| --- | --- | --- |
| (((((((((Hypertension[MeSH Terms]) OR (HTN[MeSH Terms])) OR (systolic blood pressure[MeSH Terms])) OR (diastolic blood pressure[MeSH Terms])) OR (SBP[MeSH Terms])) OR (DBP[MeSH Terms])) OR (blood pressure[MeSH Terms])) OR (Hypertension[Title/Abstract])) AND (((((((((((Child*[MeSH Terms]) OR (children[MeSH Terms])) OR (teen*[MeSH Terms])) OR (adolescent [MeSH Terms])) OR (boy*[MeSH Terms])) OR (girl[MeSH Terms])) OR (all, childhood[MeSH Terms])) OR (pediatric*[MeSH Terms])) OR (youth[MeSH Terms])) OR (teenager[MeSH Terms])) OR (toddler*[MeSH Terms]))) AND ((((((((((((sedentary behav*[MeSH Terms]) OR (screen time[MeSH Terms])) OR (sitting time[MeSH Terms])) OR (sitting time[MeSH Terms])) OR (television view*[MeSH Terms])) OR (watching television[MeSH Terms])) OR (computer use[MeSH Terms])) OR (internet use[MeSH Terms])) OR (smart phone[MeSH Terms])) OR (video game*[MeSH Terms])) OR (electronic game*[MeSH Terms])) OR (depress*[MeSH Terms])) | PubMed | 915 |
|  | Scopus | 1647 |
|  | Embase | 259 |

| **ARHQ Methodology Checklist items for Cross-Sectional study** | **Solomon-Moore E [1]** | **Pedersen J**  **[2]** | **Zou, Y [3]** | **Oliveira L [4]** | **Karatzi, K.**  **[5]** | **Barstad, LH [6]** | **Wyszyńska J [7]** | **Gui ZH**  **[8]** | **Cureau FV [9]** | **Safiri S**  **[10]** | **Christofaro**  **DGD [11]** | **BerentzenNE [12]** |
| --- | --- | --- | --- | --- | --- | --- | --- | --- | --- | --- | --- | --- |
| 1) Define the source of information (survey, record review) | ⊕ | ⊕ | ⊕ | ⊕ | ⊕ | ⊕ | ⊕ | ⊕ | ⊕ | ⊕ | ⊕ | ⊕ |
| 2) List inclusion and exclusion criteria for exposed and unexposed subjects (cases and controls) or refer to previous publications | ⊕ | ⊕ | ⊕ | ⊕ | ⊕ | ⊕ | ⊕ | ⊕ | ⊕ | ⊕ | U | - |
| 3) Indicate time period used for identifying patients | ⊕ | _ | ⊕ | ⊕ | ⊕ | ⊕ | **-** | **-** | ⊕ | ⊕ | **-** | ⊕ |
| 4) Indicate whether or not subjects were consecutive if not population-based | ⊕ | ⊕ | ⊕ | ⊕ | - | ⊕ | ⊕ | ⊕ | ⊕ | ⊕ | ⊕ | ⊕ |
| 5) Indicate if evaluators of subjective components of study were masked to other aspects of the status of the participants | U | U | U | U | U | U | U | U | U | U | U | U |
| 6) Describe any assessments undertaken for quality assurance purposes (e.g., test/retest of primary outcome measurements) | U | U | ⊕ | ⊕ | U | - | U | - | - | U | U | U |
| 7) Explain any patient exclusions from analysis | ⊕ | _ | - | ⊕ | - | ⊕ | ⊕ | - | ⊕ | ⊕ | U | U |
| 8) Describe how confounding was assessed and/or controlled. | ⊕ | ⊕ | - | ⊕ | ⊕ | - | ⊕ | ⊕ | ⊕ | ⊕ | ⊕ | ⊕ |
| 9) If applicable, explain how missing data were handled in the analysis | ⊕ | ⊕ | - | ⊕ | U | ⊕ | U | - | ⊕ | - | U | ⊕ |
| 10) Summarize patient response rates and completeness of data collection | ⊕ | ⊕ | ⊕ | ⊕ | ⊕ | ⊕ | ⊕ | ⊕ | ⊕ | - | ⊕ | ⊕ |
| 11) Clarify what follow-up, if any, was expected and the percentage of patients for which incomplete data or follow-up was obtained | ⊕ | ⊕ | U | U | - | - | U | - | U | U | - | ⊕ |

**Sup. Table 3.** Agency for Healthcare Research and Quality (AHRQ) checklist to assess quality of the cross-sectional studies *

**Sup. Table 3.** Continued

| **ARHQ Methodology Checklist items for Cross-Sectional study** | **Stamatakis E [13]** | **Berendes A [14]** | **Byun W [15]** | **Carson V [16]** | **Ullrich-French SC**  **[17]** | **Hardy LL**  **[18]** | **Lazarou C**  **[19]** | **Pardee PE [20]** |
| --- | --- | --- | --- | --- | --- | --- | --- | --- |
| 1) Define the source of information (survey, record review) | ⊕ | ⊕ | ⊕ | ⊕ | ⊕ | ⊕ | ⊕ | ⊕ |
| 2) List inclusion and exclusion criteria for exposed and unexposed subjects (cases and controls) or refer to previous publications | ⊕ | ⊕ | - | - | ⊕ | U | - | - |
| 3) Indicate time period used for identifying patients | ⊕ | ⊕ | ⊕ | ⊕ |  | ⊕ | ⊕ | ⊕ |
| 4) Indicate whether or not subjects were consecutive if not population-based | ⊕ | ⊕ | ⊕ | ⊕ | ⊕ | ⊕ | ⊕ | ⊕ |
| 5) Indicate if evaluators of subjective components of study were masked to other aspects of the status of the participants | U | U | U | - | U | U | U | U |
| 6) Describe any assessments undertaken for quality assurance purposes (e.g., test/retest of primary outcome measurements) | ⊕ | **-** | ⊕ | - | - |  | - | - |
| 7) Explain any patient exclusions from analysis | ⊕ | - | ⊕ | ⊕ | ⊕ | ⊕ | ⊕ | ⊕ |
| 8) Describe how confounding was assessed and/or controlled. | ⊕ | ⊕ | ⊕ | ⊕ | ⊕ | ⊕ | ⊕ | ⊕ |
| 9) If applicable, explain how missing data were handled in the analysis | ⊕ | **-** | ⊕ | ⊕ | - | ⊕ | - | ⊕ |
| 10) Summarize patient response rates and completeness of data collection | ⊕ | ⊕ | ⊕ | ⊕ | ⊕ | ⊕ | ⊕ | ⊕ |
| 11) Clarify what follow-up, if any, was expected and the percentage of patients for which incomplete data or follow-up was obtained | - | - | - | - | ⊕ |  | - | - |

* The items were scored as follows: if the answer were “YES,” the score was “1” and if the answers were “NO” or “UNCLEAR”, the score was “0”. The final quality scores were: low quality = 0–3; moderate quality = 4–7 and high quality ≥ 8. There was no quality criteria for inclusion of the studies in the current meta-analysis

**Sup. Table 4. Certainly of evidence assessment using the Grading of Recommendations Assessment, Development, and
Evaluation (GRADE) criteria**

| **Certainty assessment** | | | | | | | **№ of patients** | | **Effect** | | **Certainty** | **Importance** |
| --- | --- | --- | --- | --- | --- | --- | --- | --- | --- | --- | --- | --- |
| **№ of studies** | **Study design** | **Risk of bias** | **Inconsistency** | **Indirectness** | **Imprecision** | **Other considerations** | **Those with the highest screen time** | **those with the lowest screen time** | **Relative (95% CI)** | **Absolute (95% CI)** |  |  |
| **Hypertension** | | | | | | | | | | | | |
| 15 | observational studies | not serious | Serious ^a^ | not serious | not serious | strong association all plausible residual confounding would reduce the demonstrated effect dose response gradient | 2345/136605 (1.7%) | 2312/136605 (1.7%) | **OR 1.153** (1.076 to 1.234) | **3 more per 1,000** (from 1 more to 4 more) | ⨁⨁⨁⨁ High | CRITICAL |
|  |  |  |  |  |  |  |  | 0.0% |  | **0 fewer per 1,000** (from 0 fewer to 0 fewer) |  |  |
| **High systolic blood pressure** | | | | | | | | | | | | |
| 5 | observational studies | not serious | Serious ^b^ | not serious | not serious | strong association all plausible residual confounding would reduce the demonstrated effect | 2345/8651 (27.1%) | 3454/8651 (39.9%) | **WMD 1.898** (0.181 to 3.616) | **-- per 1,000** (from -- to --) | ⨁⨁⨁◯ Moderate | CRITICAL |
|  |  |  |  |  |  |  |  | 0.0% |  | **-- per 1,000** (from -- to --) |  |  |
| **High diastolic blood pressure** | | | | | | | | | | | | |
| 4 | observational studies | not serious | Serious ^c^ | not serious | Serious ^d^ | strong association all plausible residual confounding would reduce the demonstrated effect | 2165/8429 (25.7%) | 3212/8429 (38.1%) | **WMD 1.420** (-0.383 to 3.223) | **-- per 1,000** (from -- to --) | ⨁⨁◯◯ Low | CRITICAL |
|  |  |  |  |  |  |  |  | 0.0% |  | **-- per 1,000** (from -- to --) |  |  |
| **High screen time** | | | | | | | | | | | | |
| 11 | observational studies | not serious | serious^e^ | not serious | not serious | none | 2343/8645 (27.1%) | 2343/8645 (27.1%) | **WMD 0.791** (0.015 to 1.566) | **-- per 1,000** (from -- to --) | ⨁◯◯◯ Very low | CRITICAL |
|  |  |  |  |  |  |  |  | 0.0% |  | **-- per 1,000** (from -- to --) |  |  |

**CI:** confidence interval; **OR:** odds ratio; HTN, hypertension; SBP, systolic blood pressure; DBP, diastolic blood pressure; ST, screen time; CI, confidence interval; OR, odds ratio; WMD, weighted mean difference; the results are obtained from GRADE pro GDT software; Studies were classified as: ⊕⊕⊕⊕= high quality; ⊕⊕⊕◯ = moderate quality; ⊕⊕◯◯ = low quality; ⊕◯◯◯ = very low quality

#### Explanations

a. Serious inconsistency since I2 = 83.2%, P-het <0.001, which was largely unexplained in pre-specified subgroup. Downgraded

b. Serious inconsistency since I2 = 83.4%, P-het <0.001, which was largely unexplained in the pre-specified subgroup. Downgraded

c. Serious inconsistency since I2 = 89.3%, P-het <0.001, which was largely unexplained in the pre-specified subgroup. Downgraded

d. The 95% confidence interval includes no effect and the lower bound of -0.38 DBP difference is of questionable clinical importance.

e. Serious inconsistency since I2 = 92.8%, P-het <0.001, which was largely unexplained in the pre-specified subgroup. Downgraded

A B

**
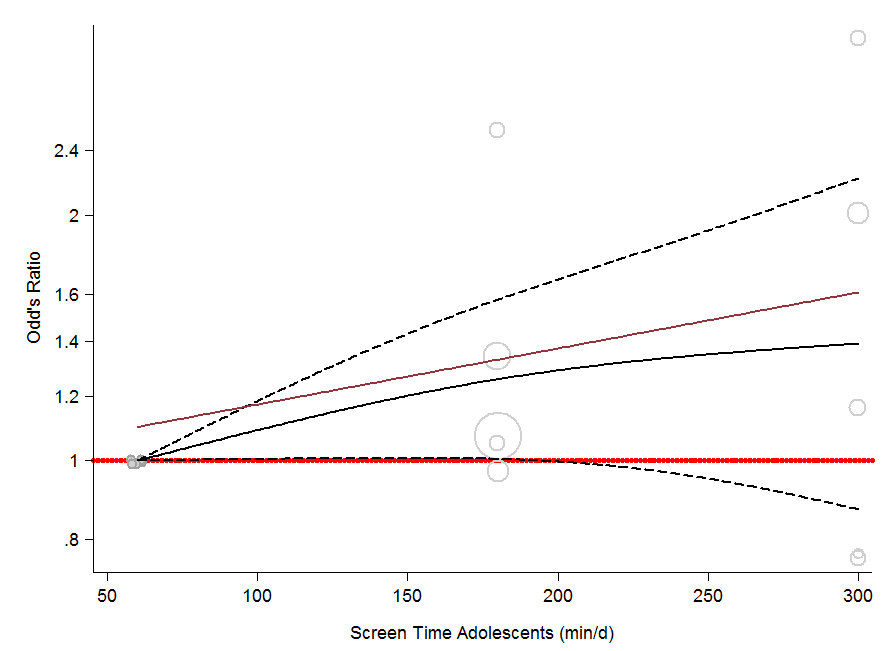
** **
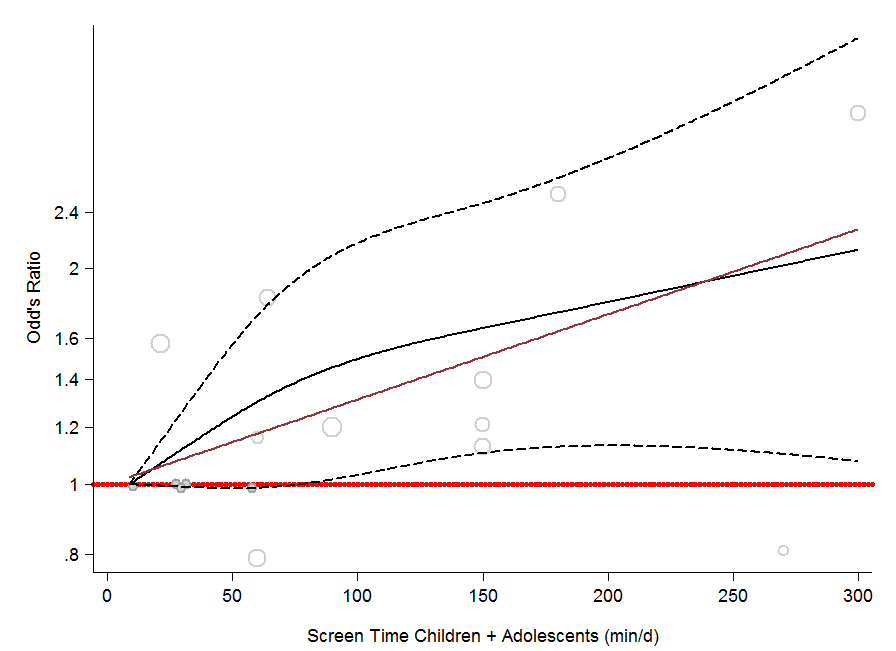
**

**C**

**
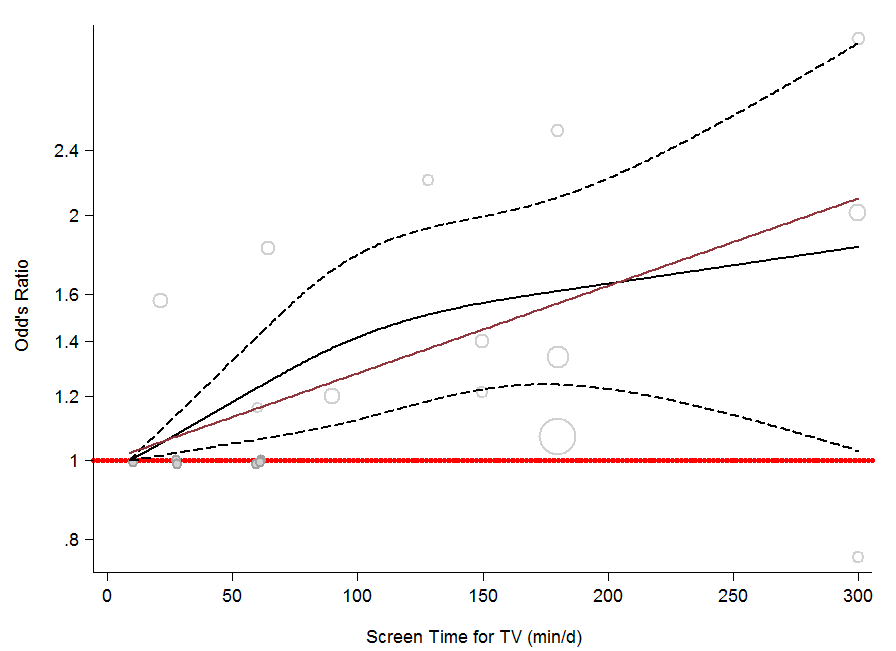
**

**Sup. Figure 2.** Dose–response association between screen time and odds of hypertension. Linear relation (solid line) and 95% CI (dashed lines) of pooled OR of HTN prevalence by 1 *min*/*day* increment of screen time of (A) adolescents (P-nonlinearity = 0.3435), (B) children + adolescents (P-nonlinearity = 0.3237) (C) TV (p- nonlinearity = 0.295).

**A B**


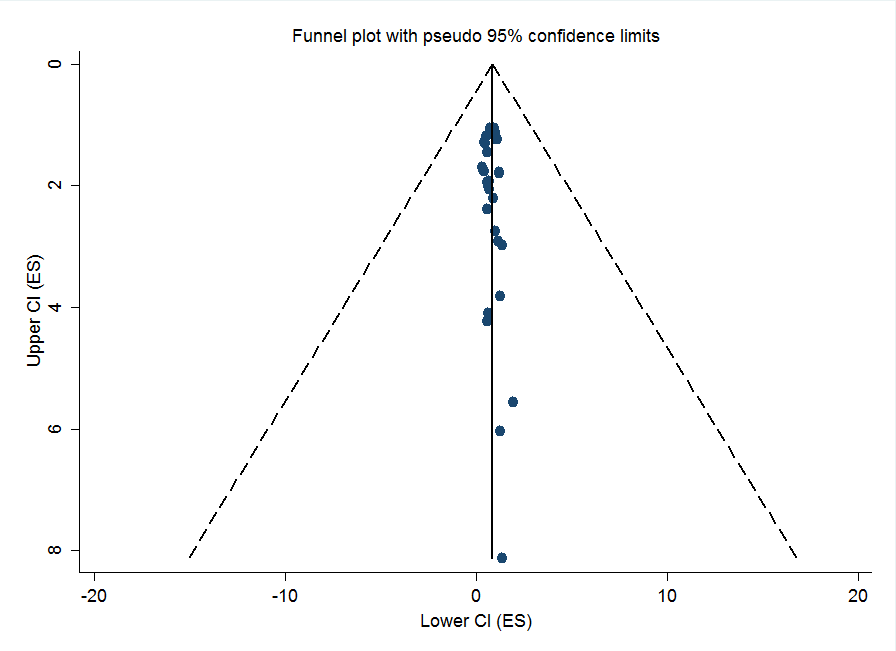

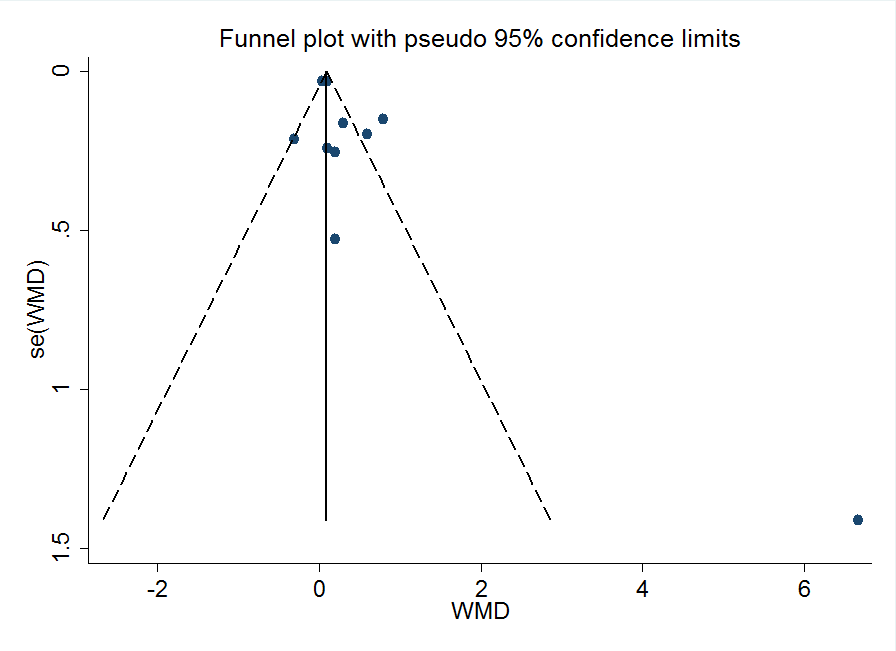


**
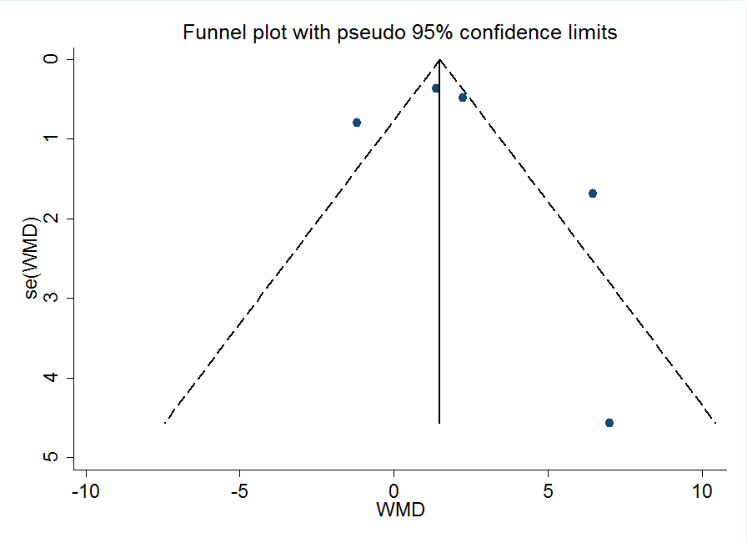
C D**
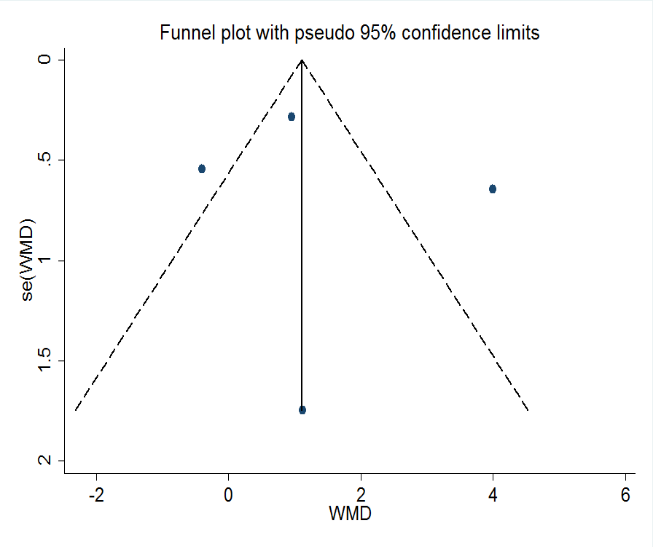


**Sup. Figure 2**. Begg's funnel plot (with pseudo 95% CIs) of the odds ratios of the association between screen time and hypertension among children and adolescents (A); the comparison of screen time between hypertensive and normotensive youth (B), the comparison of SBP between highest and lowest screen time categories (C), the comparison of DBP between highest and lowest screen time categories (D). [[OR of HTN and screen time: Egger’s test (P = 0.811) and Begg’s test (P = 0.800); SBP in highest versus lowest screen time category: Egger’s test (P = 0.600) and Begg’s test (P = 0.624); DBP in highest versus lowest screen time category: Egger’s test (P = 0.771) and Begg’s test (P = 0.497); ST in hypertensive versus normotensive youth: Egger’s test (P = 0.072) and Begg’s test (P = 0.435)].

**References**

1. Solomon-Moore, E., R. Salway, L. Emm-Collison, J.L. Thompson, S.J. Sebire, D.A. Lawlor, R. Jago, *Associations of body mass index, physical activity and sedentary time with blood pressure in primary school children from south-west England: A prospective study.* PLoS One, 2020. 15(4): p. e0232333.

2. Pedersen, J., M.G. Rasmussen, M. Neland, A. Grøntved, *Screen-based media use and blood pressure in preschool-aged children: A prospective study in the Odense Child Cohort.* Scandinavian journal of public health, 2020: p. 1403494820914823.

3. Zou, Y., N. Xia, Y. Zou, Z. Chen, Y. Wen, *Smartphone addiction may be associated with adolescent hypertension: a cross-sectional study among junior school students in China.* BMC Pediatr, 2019. 19(1): p. 310.

4. Oliveira, L., R.M. Ritti-Dias, B.Q. Farah, D.G.D. Christofaro, M.V.G. Barros, P.R.B. Diniz, F. Guimarães, *Does the type of sedentary behaviors influence blood pressurein adolescents boys and girls? A cross-sectional study.* Cien Saude Colet, 2018. 23(8): p. 2575-2585.

5. Karatzi, K., G. Moschonis, S. Botelli, O. Androutsos, G.P. Chrousos, C. Lionis, Y. Manios, *Physical activity and sedentary behavior thresholds for identifying childhood hypertension and its phenotypes: The Healthy Growth Study.* Journal of the American Society of Hypertension, 2018. 12(10): p. 714-722.

6. Barstad, L.H., P.B. Júlíusson, L.K. Johnson, J.K. Hertel, S. Lekhal, J. Hjelmesæth, *Gender-related differences in cardiometabolic risk factors and lifestyle behaviors in treatment-seeking adolescents with severe obesity.* BMC Pediatrics, 2018. 18(1).

7. Wyszyńska, J., J. Podgórska-Bednarz, K. Dereń, A. Mazur, *The Relationship between Physical Activity and Screen Time with the Risk of Hypertension in Children and Adolescents with Intellectual Disability.* BioMed Research International, 2017. 2017.

8. Gui, Z.H., Y.N. Zhu, L. Cai, F.H. Sun, Y.H. Ma, J. Jing, Y.J. Chen, *Sugar-sweetened beverage consumption and risks of obesity and hypertension in chinese Children and adolescents: A national cross-sectional analysis.* Nutrients, 2017. 9(12).

9. Cureau, F.V., U. Ekelund, K.V. Bloch, B.D. Schaan, *Does body mass index modify the association between physical activity and screen time with cardiometabolic risk factors in adolescents? Findings from a country-wide survey.* Int J Obes (Lond), 2017. 41(4): p. 551-559.

10. Safiri, S., R. Kelishadi, M. Qorbani, A. Abbasi-Ghah-Ramanloo, M.E. Motlagh, G. Ardalan, G. Shafiee, Z. Ahadi, M. Sanaei, H. Asayesh, B. Larijani, R. Heshmat, *Screen time and its relation to cardiometabolic risk among children and adolescents: The CASPIAN-III study.* Iranian Journal of Public Health, 2015. 44(1): p. 35-44.

11. Christofaro, D.G.D., S.M. De Andrade, J.R. Cardoso, A.E. Mesas, J.S. Codogno, R.A. Fernandes, *High blood pressure and sedentary behavior in adolescents are associated even after controlling for confounding factors.* Blood Pressure, 2015. 24(5): p. 317-323.

12. Berentzen, N.E., H.A. Smit, L. Van Rossem, U. Gehring, M. Kerkhof, D.S. Postma, H.C. Boshuizen, A.H. Wijga, *Screen time, adiposity and cardiometabolic markers: Mediation by physical activity, not snacking, among 11-year-old children.* International Journal of Obesity, 2014. 38: p. 1317-1323.

13. Stamatakis, E., N. Coombs, R. Jago, A. Gama, I. Mourão, H. Nogueira, V. Rosado, C. Padez, *Type-specific screen time associations with cardiovascular risk markers in children.* American Journal of Preventive Medicine, 2013. 44(5): p. 481-488.

14. Berendes, A., T. Meyer, M. Hulpke-Wette, C. Herrmann-Lingen, *Association of elevated blood pressure with low distress and good quality of life: results from the nationwide representative German Health Interview and Examination Survey for Children and Adolescents.* Psychosom Med, 2013. 75(4): p. 422-8.

15. Byun, W., M. Dowda, R.R. Pate, *Associations between screen-based sedentary behavior and cardiovascular disease risk factors in Korean youth.* Journal of Korean Medical Science, 2012. 27(4): p. 388-394.

16. Carson, V., I. Janssen, *Volume, patterns, and types of sedentary behavior and cardio-metabolic health in children and adolescents: a cross-sectional study.* BMC public health, 2011. 11: p. 274.

17. Ullrich-French, S.C., T.G. Power, K.B. Daratha, R.C. Bindler, M.M. Steele, *Examination of adolescents' screen time and physical fitness as independent correlates of weight status and blood pressure.* J Sports Sci, 2010. 28(11): p. 1189-96.

18. Hardy, L.L., E. Denney-Wilson, A.P. Thrift, A.D. Okely, L.A. Baur, *Screen time and metabolic risk factors among adolescents.* Archives of Pediatrics and Adolescent Medicine, 2010. 164(7): p. 643-649.

19. Lazarou, C., D.B. Panagiotakos, A.L. Matalas, *Lifestyle factors are determinants of children's blood pressure levels: the CYKIDS study.* J Hum Hypertens, 2009. 23(7): p. 456-63.

20. Pardee, P.E., G.J. Norman, R.H. Lustig, D. Preud'homme, J.B. Schwimmer, *Television viewing and hypertension in obese children.* Am J Prev Med, 2007. 33(6): p. 439-43.
